# Effects of appearance and gender on pre-touch proxemics in virtual reality

**DOI:** 10.3389/fpsyg.2023.1195059

**Published:** 2023-06-09

**Authors:** Mitsuhiko Kimoto, Yohei Otsuka, Michita Imai, Masahiro Shiomi

**Affiliations:** ^1^Interaction Science Laboratories, ATR, Kyoto, Japan; ^2^Faculty of Science and Technology, Keio University, Yokohama, Japan

**Keywords:** proxemics, virtual reality, social touch, pre-touch, human-agent interaction, virtual agent, personal space, gender swap

## Abstract

Virtual reality (VR) environments are increasingly popular for various applications, and the appearance of virtual characters is a critical factor that influences user behaviors. In this study, we aimed to investigate the impact of avatar and agent appearances on pre-touch proxemics in VR. To achieve this goal, we designed experiments utilizing three user avatars (man/woman/robot) and three virtual agents (man/woman/robot). Specifically, we measured the pre-touch reaction distances to the face and body, which are the distances at which a person starts to feel uncomfortable before being touched. We examined how these distances varied based on the appearances of avatars, agents, and user gender. Our results revealed that the appearance of avatars and agents significantly impacted pre-touch reaction distances. Specifically, those using a female avatar tended to maintain larger distances before their face and body to be touched, and people also preferred greater distances before being touched by a robot agent. Interestingly, we observed no effects of user gender on pre-touch reaction distances. These findings have implications for the design and implementation of VR systems, as they suggest that avatar and agent appearances play a significant role in shaping users’ perceptions of pre-touch proxemics. Our study highlights the importance of considering these factors when creating immersive and socially acceptable VR experiences.

## 1. Introduction

With the popularization of VR devices and applications, people have more opportunities for interacting with others in VR environments. Various devices and algorithms have introduced haptic feedback in VR in addition to audiovisual feedback (Van Erp and Toet, 2015; Huisman, 2016; Zenner and Kruger, 2017; Whitmire et al., 2018; Preechayasomboon and Rombokas, 2021; Kruijff et al., 2022; Kudry and Cohen, 2022; Nunez et al., 2022; Shell et al., 2022; Villa et al., 2022). Due to such devices and algorithms, humans are more often haptically interacting with virtual characters, and human behavior during touch interactions in VR is being actively studied (Bailenson and Yee, 2008; Huisman et al., 2014; Hoppe et al., 2020; Sykownik and Masuch, 2020; Gallace and Girondini, 2022).

In VR, the appearances of virtual characters affect the perceptions and behaviors of others. Such appearance-related effects on people’s perceptions and behaviors are being investigated when they touch others and/or when they themselves are touched. Bailenson and Yee (2008) reported that people touched a human-like virtual character with less force than when they touched a cylindrical object (non-human object); they touched a virtual character’s face with less force than its torso. These studies, which focused on the behaviors and perceptions of humans when they touch and are touched, provide knowledge about creating virtual agents that behave in a human-like manner when touching and/or being touched. Here, in this paper, we define an agent as a computer-controlled virtual character.

However, few studies focused on the situation before humans are touched. In the fields of computer interface and manipulation in robotics, some studies have addressed pre-touch situations (Hinckley et al., 2016; Aslan and André, 2017; Lancaster et al., 2017; Müller et al., 2019). For example, Lancaster et al. (2017) presented a novel framework combining pre-touch sensing and deep learning for more accurate and efficient object pose estimation. Hinckley et al. (2016) investigated the emerging pre-touch modality *via* a self-capacitance touchscreen that can sense multiple fingers above a mobile device and grip around the screen’s edges. In contrast, in the context of interaction between humans and virtual characters/robots, there are few studies about pre-touch interaction.

Touch interaction begins before physical contact; such pre-touch interaction is also related to representations of human-likeness (Shiomi et al., 2018; Mejía et al., 2021a, 2023). For example, before being touched at a certain distance, humans respond by turning toward the approaching hand and maintaining a distance from it. In a human-robot interaction study, Shiomi et al. (2018) reported that humans prefer a robot that responds to an approaching hand at a modeled comfortable distance, based on pre-touch interactions between humans. In a VR study, Mejía et al. investigated the distance at which humans begin to feel uncomfortable when a virtual hand approaches their face (called the pre-touch reaction distance) and reported that pre-touch reaction distances to the face measured in VR were close to those measured in physical space (Mejía et al., 2021a).

Although Mejía et al. (2021a) concluded that pre-touch reaction distances in VR resemble those in physical environments, the effects of VR’s characteristic nature on pre-touch reaction distances are not well investigated. In particular, previous works did not consider the embodiment of the avatar. Instead, participants simply looked at visual stimuli without much awareness of their body. Here, in this paper, we define an avatar as a human-controlled virtual character. Avatar embodiment influences interpersonal spaces in VR (Yee and Bailenson, 2007; Zibrek et al., 2020; Rivu et al., 2021; Buck et al., 2022). Yee and Bailenson (2007) described that participants using a high attractiveness avatar walked closer to another avatar than to a low attractiveness avatar. Rivu et al. (2021) reported that users’ preferred interpersonal spaces between friends increased when the gender of an avatar was different from their own. These studies suggest that avatar appearance and user gender affect the proximity between agents and avatars. Unfortunately, such effects on pre-touch reaction distances have not been investigated.

In this study, our primary goal is to measure pre-touch reaction distances in VR, considering the influence of appearance and gender as crucial factors. We examine the effects of avatar appearance, agent appearance, and user gender on these distances. Drawing on prior research in proxemics, we have formulated hypotheses about the impact of avatar, agent, and gender factors. Several studies demonstrated that women generally maintain a larger interpersonal space than men (Bailenson et al., 2001; Iachini et al., 2014; Zibrek et al., 2020, 2022; Rapuano et al., 2021).

*Hypothesis 1-1:* Women will maintain greater pre-touch reaction distances than men.

Furthermore, when the avatar is embodied, its appearance may also influence pre-touch reaction distances.

*Hypothesis 1-2:* People using a female avatar will maintain greater pre-touch reaction distances than those using a male or a robot avatar.

The appearance of the agent is another factor that can affect interpersonal space in virtual reality. Multiple studies have shown that people tend to maintain larger distances from male agents (Wieser et al., 2010; Iachini et al., 2016; Huang et al., 2022; Zibrek et al., 2022), and prefer larger distances from non-human objects compared to human-like agents (Iachini et al., 2014; Huang et al., 2022). These effects may also be observed in pre-touch reaction distances.

*Hypothesis 2:* People will maintain greater pre-touch reaction distances when reacting to an approaching hand of a male or a robot agent than of a female agent.

To achieve the goal, we experimentally measured pre-touch distances to an avatar’s face and body, systematically varying the appearance of avatars and agents using man, woman, and robot-like depictions. We also examined the role of user gender and how different combinations of avatar/agent appearances and user gender affected pre-touch reaction distances in VR.

## 2. Related work

### 2.1. Proxemics in VR environments

Humans generally maintain a certain distance from others. Hall’s seminal work on proxemics (Hall, 1966) has inspired many researchers to study interpersonal space not only between humans but also between a human and a virtual character. Humans also maintain a certain distance from a virtual character in VR environments (Bailenson et al., 2001, 2003; Wilcox et al., 2006; Llobera et al., 2010; Huang et al., 2022). Huang et al. (2022) investigated proxemics when humans interact with virtual agents in augmented reality. Li et al. (2019) compared human-robot proxemics between VR and physical environments and concluded that humans maintained larger distances from a virtual robot than a physical robot. Such social factors as emotional expressions (Ruggiero et al., 2017; Bönsch et al., 2018, 2020), motion attractiveness (Zibrek et al., 2020), gaze (Bailenson et al., 2001, 2003), and coughing behaviors (Shiomi et al., 2022) affect proxemics in human-agent interactions as well as in human-human interactions in physical environments. Based on the foundation of proxemics, various works have proposed designs for virtual and robotic behaviors (Althaus et al., 2004; Kirby et al., 2009; Svenstrup et al., 2010; Novick et al., 2018). These works show how proxemics also affects human-agent and human-human interaction, and knowledge of proxemics is critical for designing acceptable pre-interaction behaviors of agents.

In contrast to the vast background of proxemics on positions between humans as well as between humans and agents, few studies have focused on proxemics in touch interaction, which addresses the close distance maintained by humans to an approaching hand. Concerning work on proxemics in touch interaction, in the field of human-robot interaction, few researchers have investigated pre-touch reaction distances (Shiomi et al., 2018; Mejía et al., 2021b). Both of these works also measured pre-touch reaction distances in VR and designed virtual agents that react to an approaching hand based on the measured distance at which humans felt uncomfortable (Mejía et al., 2021a). Although Mejía et al. measured pre-touch reaction distances in VR, since they focused on the face without considering the presence of the avatar’s body, how avatar embodiment affects the pre-touch reaction distance in VR remains unclarified.

### 2.2. Effects of appearance and user’s gender on proxemics in VR environments

Human perceptions of others are greatly influenced by appearance and attractiveness, whether in physical interactions or virtual environments. According to Dion et al. (1972), physically attractive individuals are often perceived as possessing more socially desirable personality traits and leading better lives than their less attractive counterparts, and this relationship between attractiveness and perceived goodness was further validated by Gross and Crofton (1977). Principe and Langlois (2011) found that less attractive faces evoke greater disgust and negative affect than more attractive faces, emphasizing the substantial role attractiveness plays in eliciting affective responses.

In the interactions between humans and virtual characters, the influence of attractiveness and appearance also influences human perceptions and behaviors. Waddell and Ivory (2015) conducted a field experiment to examine how avatar attractiveness, avatar gender, and user gender interact to influence responses to a requested favor. The study found that attractive avatars received more help than less attractive avatars. However, female users received less help than male users when represented by avatars that were less attractive or men. Nowak and Rauh (2005) examined how avatar characteristics, such as their androgyny and anthropomorphism, influence online users’ perceptions. They found that more anthropomorphic avatars were perceived as more attractive and credible, with a preference for avatars matching the user’s gender. These studies suggest the profound influence of attractiveness and appearance on human perceptions and interactions, both in physical and virtual environments.

Proxemics is also affected by the appearances of an avatar and an agent. Many works have reported the effects of appearance-related factors on proxemics, including avatar size, realism, gender, and age (Bailenson et al., 2001, 2003; Iachini et al., 2014, 2016; Zibrek et al., 2017; Buck et al., 2019, 2022; Lisi et al., 2021; Mousas et al., 2021; Rivu et al., 2021; Huang et al., 2022). Some works reported the effects of non-human-like appearances on proxemics (Iachini et al., 2014; Mousas et al., 2021; Huang et al., 2022). For example, Iachini et al. (2014) reported that humans maintained a larger distance from a cylindrical object than a human-like agent.

Gender is another factor that has been scrutinized. An agent’s gender influences the interpersonal distance between users and agents. Some studies have shown a similar tendency where people keep a larger distance from male agents (Wieser et al., 2010; Iachini et al., 2016; Huang et al., 2022; Zibrek et al., 2022). The gender of the user is also related to proxemics in VR. Some studies showed that women prefer a larger distance than men (Bailenson et al., 2001; Iachini et al., 2014; Zibrek et al., 2020, 2022; Rapuano et al., 2021).

Another factor is an avatar’s embodiment. Studies in non-proxemics contexts reported the effects of gender transfer/swap on decision-making (Bolt et al., 2021), touch perception (Fusaro et al., 2021; Mello et al., 2021), gender bias (Schulze et al., 2019; Wu and Chen, 2022), and task performance (Lee et al., 2014). In the context of proxemics, few studies have investigated the effects of avatar embodiment when the user’s gender does not match the avatar’s gender (Buck et al., 2019; Rivu et al., 2021). Rivu et al. (2021) reported that the preferred distance between friends increased when users employed avatars of the another sex.

These studies suggest that the appearances of agents, avatars, and user gender affect proxemics, as does the interaction of these factors. In this study, we focus on how humans are affected by the appearances of their avatars and the agents with whom they interact as well as the gender of users during pre-touch proxemics.

## 3. Material and method

### 3.1. Conditions: appearances of avatar, agent, and user gender

We prepared three factors in our experiment: (1) avatar appearance (within-subject, three levels: man/woman/robot), (2) agent appearance (within-subject, three levels: man/woman/robot), and (3) participant gender (between-subject, two levels: man/woman). We prepared three 3D models for the appearances of the avatars and agents: man, woman, and robot. For the 3D model of the robot avatar and agent, we used Pepper, a robot developed by SoftBank Robotics. These models of avatars and virtual agents are equipped with joints that allow the avatars to mirror movements in real space and the virtual agents to move their hands toward the avatars.

### 3.2. Task design

#### 3.2.1. Overview

**Figure 1:**
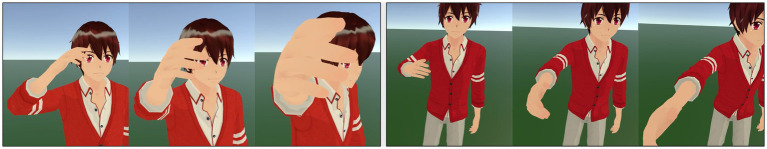
Examples of implemented behaviors of hand approaching face and body. We measured the pre-touch reaction distances to the face and body.

To investigate pre-touch proxemics, we followed related works (Shiomi et al., 2018; Mejía et al., 2021a,b) and measured the pre-touch reaction distances, which are those at which a person starts to feel uncomfortable before being touched. This measurement is based on the stop-distance procedure (Hayduk, 1985), which is the most widely used approach for calculating interpersonal distance. We measured pre-touch reaction distances to the face and body (Figure 1). We controlled the appearances of the avatar and the agent and examined the effect on pre-touch reaction distances.

#### 3.2.2. Implementation of touching behavior of agent

**Figure 2:**
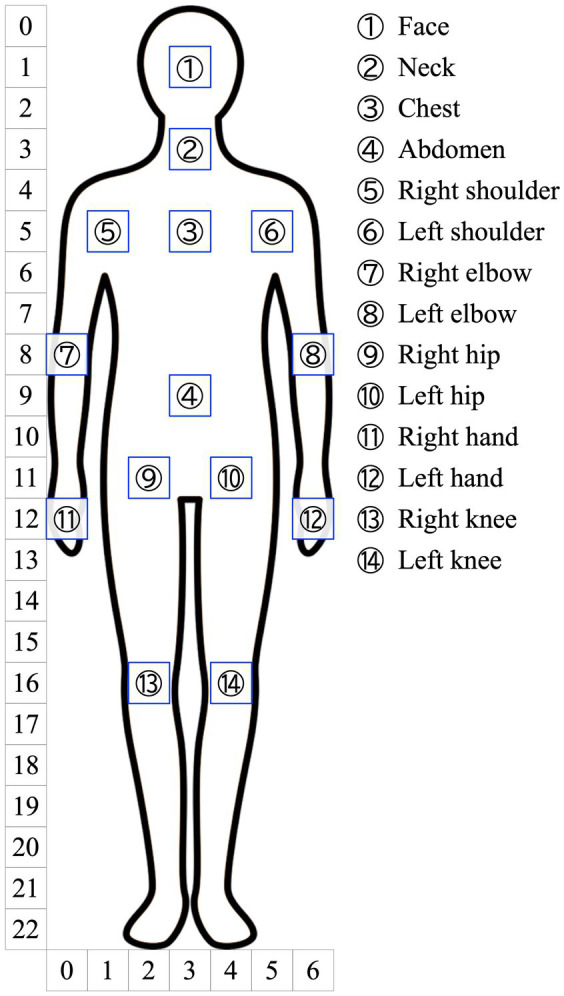
Avatar’s body parts used to the target of approach motion.

In the experiment, our agent raised its right hand to the avatar’s face or body as follows. The approach motion to the face was implemented from a total of 35 angles: combinations of five angles of ±30 degrees in 15-degree increments horizontally and seven angles of ±45 degrees in 15-degree increments vertically. The approach motion to the avatar’s body was implemented from the front at 13 points on the avatar’s body: neck, chest, abdomen, and both shoulders, elbows, hands, hips, and knees (Figure 2). The target points of the body were not recognized in real-time by a sensor but were rather pre-determined. The virtual agent’s hand was initially displayed 70 [cm] from the avatar and implemented to approach the target point at approximately 0.2 [m/s]. The agent moved its hand toward the target point in a straight line horizontally to the ground plane, while the avatar’s pose was determined using inverse kinematics.

We used the Unity game engine (Unity Technologies, version: 2019.4.20f1) to develop the virtual environments, movements of avatars, and agents. Visual stimuli were presented using a head-mounted display (HTC VIVE Pro Eye, resolution: 1440 × 1,600 pixels per eye, field of view: 100 degrees, refresh rate: 90 Hz). A computer (DELL ALIENWARE m15 R3, OS: Windows 10 Pro, RAM: 32 GB, CPU: Intel Core i9-10980HK, GPU: NVIDIA GeForce RTX 2080 SUPER Max-Q) controlled the stimuli.

#### 3.2.3. Procedure

**Figure 3:**
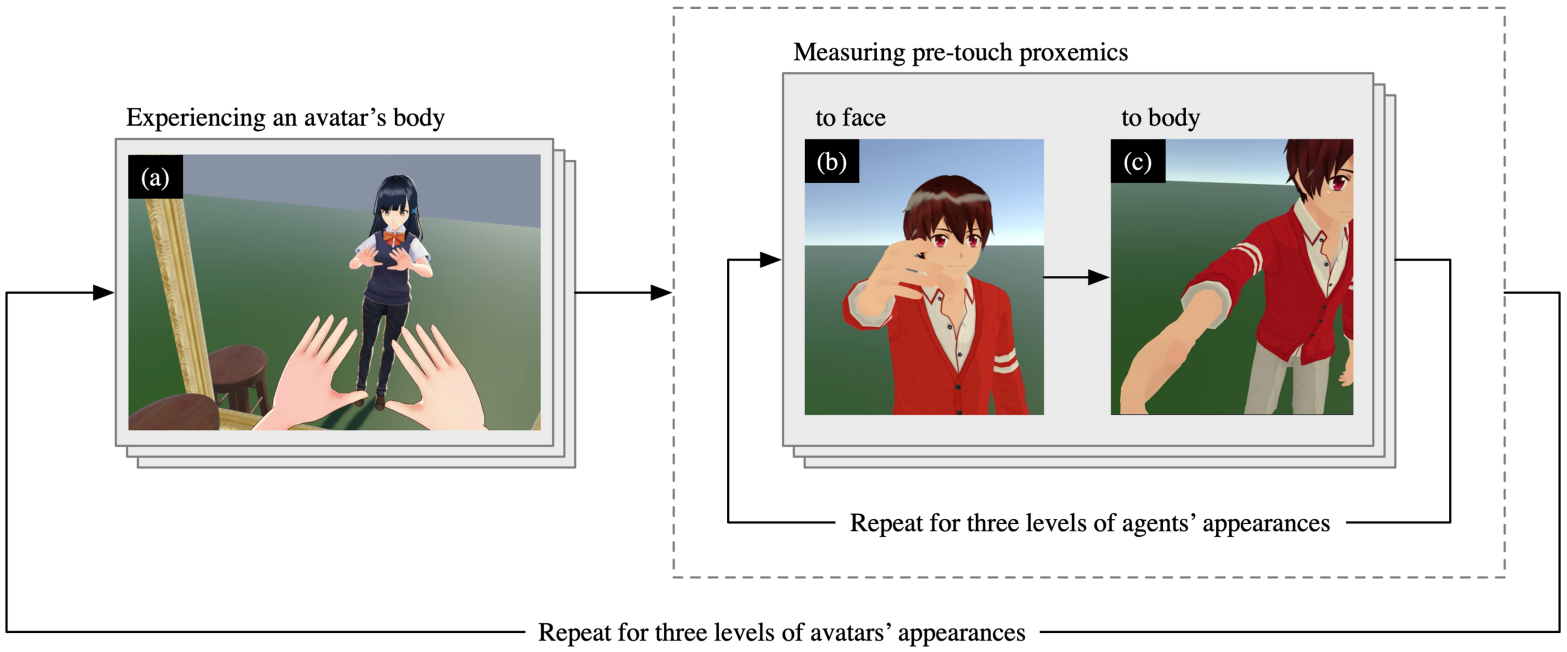
Procedure of measurement of the pre-touch reaction distance.

First, the participant received an explanation of the experiment and gave informed consent. The participants were informed about when and how to press a button on the controller during the explanation of the experiment. They then put on a head-mounted display and held controllers in both hands. The HTC VIVE Pro Eye and its controllers were used for VR stimuli and avatar control. The pre-touch reaction distances were measured based on the procedure shown in Figure 3. The measurement procedure consists of three steps: (a) avatar experience, (b) measurement of pre-touch reaction distance to the face, and (c) measurement of pre-touch reaction distance to the body. When measuring the distances to the face, participants were seated in a chair. When measuring the distances to the body, participants were standing.

First, participants experienced an avatar (man/woman/robot) to become accustomed to the avatar body in front of the mirror, following related works (Oh et al., 2016; Krekhov et al., 2019; Frisanco et al., 2022). There is no unified rule regarding the time spent on this process, as it depends on the experiment; in our case, we adopted a duration of 2 min. They could freely move its hands and head in a standing position and observed it through a mirror placed in front of the avatar (Figure 3A). The movements of the human head and arms in real space were reflected in the avatar’s movements by environmental sensors, which detected the positions of the head-mounted display and the controllers held in each hand by the participants.

After the step (a), the scene changed to display messages informing the participant that the agent would start moving its hands when they were ready. Then, participants pressed a button on the controller, and the scene changed to show the agent in front of the avatar. This scene switch was also implemented between measuring the pre-touch reaction distance for the face and body. At the beginning of the measurement, the virtual agent was displayed with its right hand in the initial position. When the participant pressed a button on the controller, the hand began to move toward the avatar’s face. If the participant felt uncomfortable, they pressed the button again, the agents stopped moving their hand, and the system measured the distance between the agent’s hand and the avatar’s face. Then, the participant pressed the button again, causing the agent to transition to the next initial position immediately. This was repeated randomly from the 35 angles described in Section 3.2.2 in a random order. During the pre-touch distance measurement to the face, the avatar’s face orientation was fixed in a frontal direction, and the hand-approach angle was identical for all participants.

Next, we measured the pre-touch reaction distance to the hand’s approaching motion to the body in the similar procedure for the face. However, the direction of the avatar’s face was not fixed, and participants were instructed to look at the hand approaching the body and were allowed to stop it when they felt uncomfortable and wanted no further approaching. The position of the avatar’s body was fixed, and the approaching motion of the hands was identical for all participants. All 13 target points on the avatar’s body was used for the measurement. The order of the pointes was randomly decided. After measuring the pre-touch reaction distance of the body, we changed the agent’s appearance and repeated the measurements of the pre-touch reaction distances of the face and body. This process was repeated with the three virtual agents. For each avatar, participants repeated steps (b) to (c) for all three agents, resulting in a total of nine conditions. We randomized the order in which participants experienced the avatars as well as the appearances of agents; however, the order of steps (b) to (c) remained consistent throughout the experiment.

All procedures were approved by the Bioethics Committee of Faculty of Science and Technology, Keio University, Application No. 2020–87.

### 3.3. Participants

Twenty participants (ten men and ten women, mean age = 22.65 years, *SD* = 1.93 years) took part in our experiments.

## 4. Results

**Figure 4:**
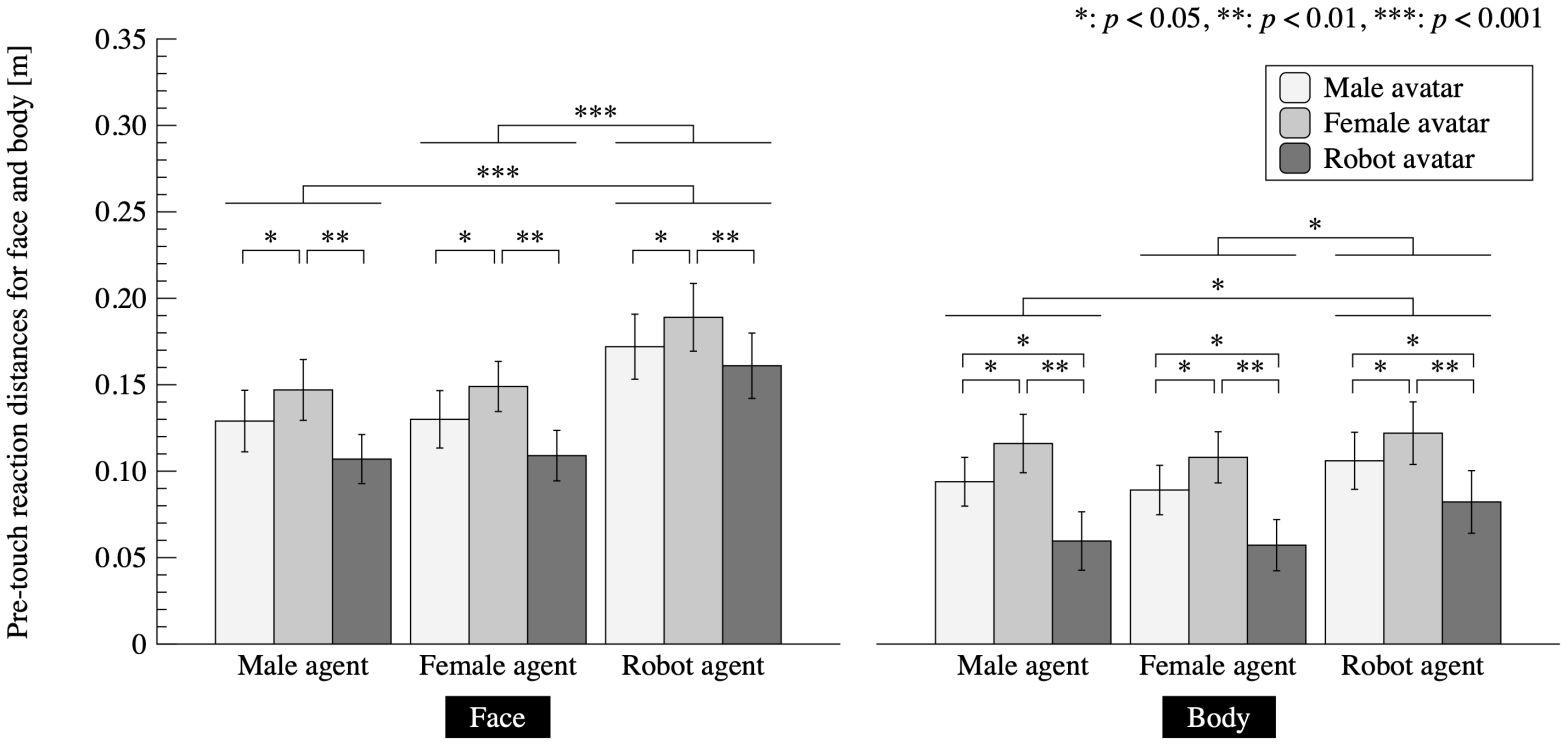
Mean pre-touch reaction distances for face and body. Error bars denote ± standard error of the mean.

In regard to pre-touch reaction distances to the face, we used the mean distances of 35 approaching angles and analyzed the data using *N* = 20, which represents the number of participants. For pre-touch reaction distances to the body, we used the mean distances of 13 points on the avatar’s body and also analyzed the data using *N* = 20. Figure 4 and Table 1 shows the pre-touch reaction distances for a face and a body. For those of a face, we conducted a three-way mixed ANOVA for the avatar, agent, and gender factors and found significant main effects in the avatar factor (*F*(2, 36) = 10.31, *p* < 0.001, partial *η*^2^ = 0.364) and the agent factor (*F*(1.47, 26.48) = 34.74, *p* < 0.001, partial *η*^2^ = 0.659). We found no significant effects in the gender factor (*F*(1, 18) = 0.830, *p* = 0.374, partial *η*^2^ = 0.044) or in the interactions between the avatar × gender factors (*F*(2, 36) = 1.16, *p* = 0.324, partial *η*^2^ = 0.061), agent × gender (*F*(2, 36) = 1.07, *p* = 0.353, partial *η*^2^ = 0.056), avatar × agent factors (*F*(4, 72) = 1.18, *p* = 0.327, partial *η*^2^ = 0.061), and avatar × agent × gender factors (*F*(4, 72) = 2.24, *p* = 0.074, partial *η*^2^ = 0.110). Multiple comparisons with Tukey’s HSD for the avatar factor revealed significant differences between conditions: female avatar > male avatar (*p* = 0.024), and female avatar > robot avatar (*p* = 0.005). Multiple comparisons with Tukey’s HSD for the agent factor showed significant differences between conditions: robot agent > female agent (*p* < 0.001) and robot agent > male agent (*p* < 0.001).

**Table 1:** Pre-touch reaction distances for the face and body, organized by avatar and agent appearances.

Target	Agent	Avatar	Mean	*SE*
Face	Man	Man	0.129	0.0178
		Woman	0.130	0.0166
		Robot	0.172	0.0188
	Woman	Man	0.147	0.0176
		Woman	0.149	0.0145
		Robot	0.189	0.0196
	Robot	Man	0.107	0.0142
		Woman	0.109	0.0146
		Robot	0.161	0.0189
Body	Man	Man	0.0939	0.0141
		Woman	0.0891	0.0143
		Robot	0.106	0.0165
	Woman	Man	0.116	0.0169
		Woman	0.108	0.0148
		Robot	0.122	0.0181
	Robot	Man	0.0596	0.00954
		Woman	0.0572	0.00901
		Robot	0.0822	0.0114

These results indicate that (1) those using a female avatar prefer larger distances before their face is touched than when using male and robot avatars, and (2) people prefer larger distances before their face is touched by a robot agent than by male and female agents.

For the pre-touch reaction distances for the body, we conducted a three-way mixed ANOVA for the avatar, agent, and gender factors and found significant main effects in the avatar factor (*F*(1.36, 24.41) = 14.251, *p* < 0.001, partial *η*^2^ = 0.442) and the agent factor (*F*(2, 36) = 5.773, *p* = 0.007, partial *η*^2^ = 0.243). We found no significant effects in the gender factor (*F*(1, 18) = 0.0210, *p* = 0.886, partial *η*^2^ = 0.001) or in the interaction between the avatar × gender factors (*F*(2, 36) = 0.213, *p* = 0.809, partial *η*^2^ = 0.012), agent × gender (*F*(2, 36) = 2.048, *p* = 0.144, partial *η*^2^ = 0.102), avatar × agent factors (*F*(4, 72) = 1.159, *p* = 0.336, partial *η*^2^ = 0.061), and avatar × agent × gender factors (*F*(4, 72) = 1.302, *p* = 0.278, partial *η*^2^ = 0.067). Multiple comparisons with Tukey’s HSD for the avatar factor revealed significant differences between conditions: male avatar > robot avatar (*p* = 0.014), female avatar > male avatar (*p* = 0.010), and female avatar > robot avatar (*p* = 0.001). Multiple comparisons with Tukey’s HSD for the agent factor showed significant differences between conditions: robot agent > male agent (*p* = 0.026) and robot agent > female agent (*p* = 0.040).

These results indicate that (1) people using a female avatar prefer larger distances before their body is touched than people using a male and a robot avatar, and (2) people using a male avatar prefer larger distances before their body is touched than people using a robot avatar. In addition, these results indicate that (3) people prefer larger distances before their body is touched by the robot agent than from male and female agents.

Thus, hypothesis 1-1 is not supported, hypothesis 1-2 is supported, and hypothesis 2 is partially supported.

## 5. Discussion

### 5.1. Implications

Our results identified strong effects of the appearance of agents and embodied avatars on pre-touch proxemics. People maintained larger pre-touch distances to the robot agent than to male and female agents. This result is partially consistent with the tendency identified by related works, which reported that people maintained larger distances to a pillar-like object than human agents (Iachini et al., 2014; Huang et al., 2022). Iachini et al. (2014) discussed that such a shorter distance to a female agent reflects attraction and self-protection mechanisms. In our experiments, participants preferred to maintain larger distances from the robot agent than from either the female or male agent. These results suggest that the robot’s appearance elicits strong avoidance perceptions during pre-touch interactions, which occur at very close distances.

Our results also showed that people maintained larger distances with female avatars than with male and robot avatars, although we did not find any significant main effects of user gender and interaction with the appearances of the agents/avatars. This distance trend with female avatars is consistent with the trend in the interpersonal distances of women, which they generally maintain greater distances than men (Bailenson et al., 2001; Iachini et al., 2014; Zibrek et al., 2020, 2022; Rapuano et al., 2021). These results could be interpreted as a greater effect of avatar gender embodiment than user gender in pre-touch proxemics. People’s behavior in VR environments is influenced by the representations of their avatars, the so-called Proteus effect (Yee and Bailenson, 2007; Ratan et al., 2020; Szolin et al., 2022). In our experiments, the embodiment of the female avatar might have induced users to engage in stronger self-protective behaviors than the embodiments of the male and robot avatars in pre-touch proxemics.

On the other hand, our results did not show significant effects of participants’ gender. In psychology, the concept of in-group advantage (Tajfel et al., 1971) suggests that individuals may feel more comfortable with members of their own group. This phenomenon could play a role in how participants react to the approaching hands of avatars and agents of different genders. Our results imply that, in the context of our study, we did not observe a clear in-group advantage effect. However, it is worth noting that the absence of significant findings in our study does not necessarily rule out the possibility of in-group advantage playing a role in other contexts or with different experimental designs. Future research with a more focused experimental design, perhaps examining the effects of various combinations of each factor step-by-step or manipulating the degree of representation of participants’ own gender within the VR environment, could help illuminate the complexities of this phenomenon, including the potential influence of in-group advantage on participants’ reactions to different avatar and agent genders. Furthermore, exploring how the degree of realism and attractiveness of avatars and agents may interact with in-group advantage could provide a more comprehensive understanding of social interactions in VR environments.

Our findings lead to the following suggestions for the pre-touch behaviors of virtual agents in VR. People felt uncomfortable at a certain distance before being touched, and this distance differed depending on the appearances of the interaction participants. To behave in a human-like manner, virtual agents should react in pre-touch situations at a distance that considers appearances. Our findings also highlight the need for carefully designing touch behaviors based on the appearances of avatars and agents. Since touching is a behavior that can easily violate personal spaces, it must be carefully designed. Our experimental results indicated that coordinating people’s acceptable distances before being touched is complicated, based on the characteristics of the interaction participants. To avoid such unsocial behavior as sexual harassment in VR (Neyret et al., 2020), touch behaviors must be designed based on the appearances of avatars/agents and the gender of their users.

### 5.2. Bodily maps of pre-touch proxemics

**Figure 5:**
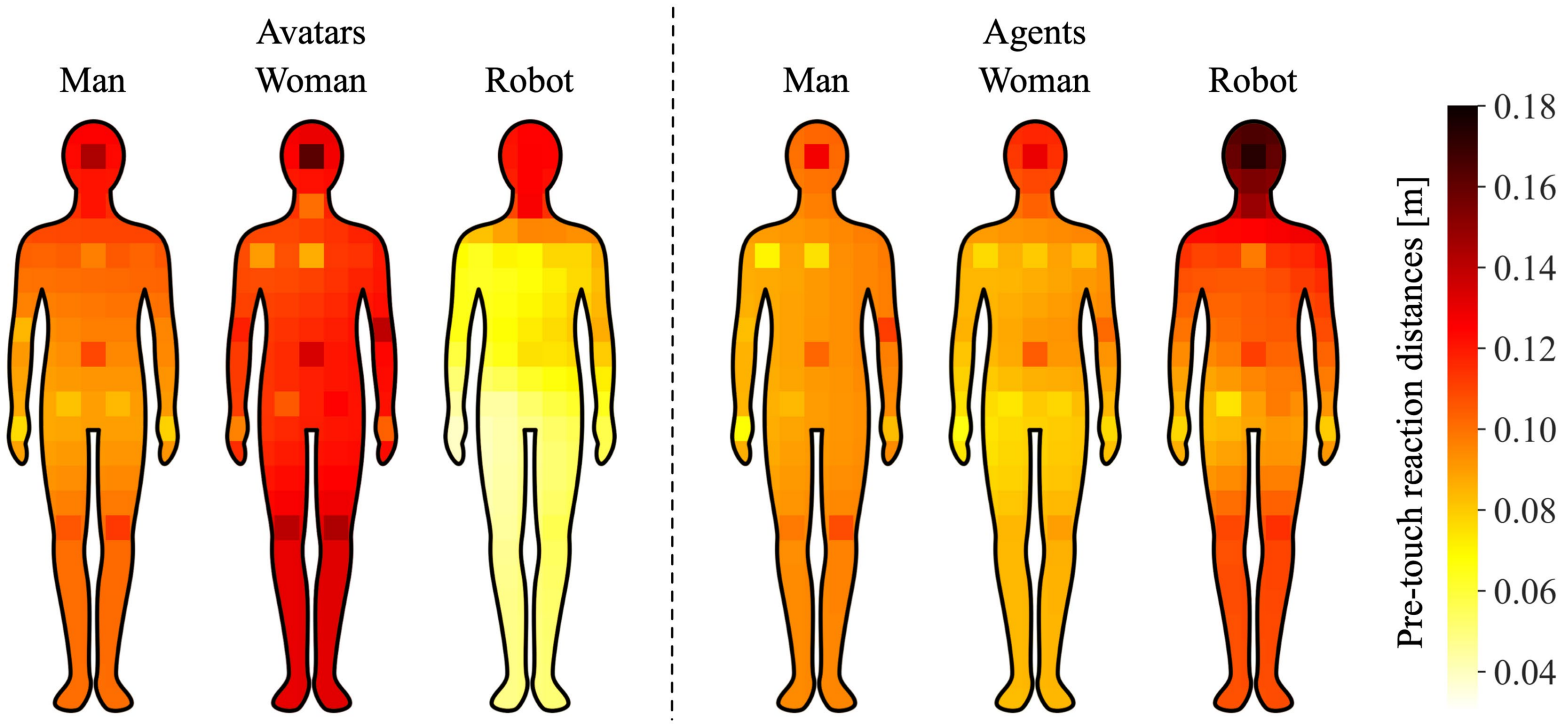
Bodily maps of pre-touch reaction distances. Color bar indicates the average and estimated pre-touch reaction distances.

We measured the pre-touch reaction distance to the face and 13 points on the body, as described in Section 3.2.2. To understand the broad tendency of pre-touch proxemics of the body, we created bodily maps of the pre-touch reaction distances, which are shown in Figure 5. These maps allow for a visual representation of the differences in distances between these points, although they do not provide conclusive results. To create the maps, we first plotted the average pre-touch reaction distances for the face and 13 points on a 7 × 23 grids, as illustrated in Figure 3. Then, we estimated the distances for the remaining points on the grid using ordinary kriging (Wackernagel, 2003), a statistical method for interpolating values at unobserved locations based on spatial correlation of observed data. Finally, we overlaid the silhouette image of a human body onto the heatmaps created based on the observed and estimated pre-touch reaction distances.

As shown in Figure 5, the effects of avatar and agent factors have a large impact on the pre-touch reaction distances. In particular, when participants used a female avatar, they tended to maintain the larger distances, while when they used a robot avatar, they tended to maintain the closer distances compared to other avatar and agent conditions. Interestingly, we found that the hands were less influenced by the avatar and agent factors than other body parts, and participants tended to maintain closer distances to hands compared to other parts. This trend is partially consistent with previous research on bodily patterns in social touch contexts (Suvilehto et al., 2015, 2019), where participants were asked to color silhouettes of human bodies in an online questionnaire to indicate where they would allow touch by individuals in different social relationships (e.g., partner, friend, stranger). Their results showed that the regions allowed to be touched by strangers were limited to the hands, shoulders, and arms, suggesting that hands are the most acceptable body parts to be touched even in VR.

These findings have implications for the design and implementation of VR systems, as they suggest that the appearance of avatars and agents can influence users’ pre-touch reaction distances to different body parts. Understanding these factors is crucial for creating more immersive and socially acceptable VR experiences.

### 5.3. Limitations

Our experiment has several limitations. First, rendering the reality of avatars and agents may affect pre-touch proxemics. Some studies have investigated its effects on the reality of agents (Jo et al., 2017; Zibrek et al., 2017, 2018). Regarding the realism of models, it has been reported that participants exhibited the highest sense of body ownership and presence when inhabiting a cartoon-like virtual avatar mimicking the participant’s outfit, rather than an avatar reconstructed from a real image of the participant’s appearance (Jo et al., 2017). Additionally, it is important to consider the concept of the Uncanny Valley (Mori et al., 2012), which suggests that as a virtual character becomes more human-like, it may evoke a sense of eeriness or discomfort in the observer. Thus, the level of perceived comfort with a virtual character may be related to its degree of realism. In our study, the cartoon characters may have been perceived as less strange or more comfortable than the robot, which may have influenced the participants’ preferences for shorter distances to the cartoon characters. At the same time, the participants in our study were affected by the appearances of both avatars and agents, making the effects of combinations of realism and attractiveness between avatars and agents more complex. Although Zibrek et al. (2017) did not find significant effects of rendering reality on proxemics in VR, they did not investigate the effects of combinations between rendering reality of avatars and agents. From the perspective of such effects of combinations, further comprehensive research is needed.

The similarity between an avatar’s appearance and the user’s body image should be addressed. The wide variety of non-human appearances of avatars/agents and their combinations should also be investigated. In our experiments, we used a humanoid robot as a non-human character and did not investigate the effects of the degree of the humanity of the avatars/agents’ appearances. Our participants were aware that they were interacting with computer agents, although in situations where people interact with others using avatars, such awareness of who controls the character of interlocutor might affect pre-touch proxemics. For example, Rivu et al. (2021) reported that people’s preferred distances between gender-swapped avatars changed when they interacted with friends. According to reports, socially close individuals were allowed to touch a wider bodily area compared to others (Suvilehto et al., 2015, 2019). For non-human avatar appearances, the familiarity of the appearance should also be addressed. In a study of human-robot interaction, Takayama and Pantofaru (2009) reported that personal experience with robots reduces the personal space around them. Such a social effect must be further studied.

## 6. Conclusion

We focused on the effects of appearances and user gender on pre-touch proxemics. We prepared different kinds of appearances of user avatars and touching agents and measured the distances at which people felt uncomfortable before being touched. Our experimental results showed that they changed their pre-touch reaction distances based on the appearances of the avatars and the agent; we observed no effects of user gender. Overall, people using a female avatar tended to maintain greater distances prior to being touched compared to those using male and robot avatars, while people tended to accept closer approaching by human-like agents than by robots prior to being touched. Our results suggest that people’s perceptions of pre-touch situations are affected by the appearances of avatars and agents and less by user gender. These findings pose implications for the design of human-like behavior of virtual agents when responding to touch, e.g., changing the pre-touch reaction distance.

## Data availability statement

The raw data supporting the conclusions of this article will be made available by the authors, without undue reservation.

## Ethics statement

The studies involving human participants were reviewed and approved by Bioethics Committee of Faculty of Science and Technology, Keio University, Application No. 2020-87. The patients/participants provided their written informed consent to participate in this study.

## Author contributions

MK, MI, and MS contributed to conception and design of the study. MK and YO developed the software, and performed the data curation and statistical analysis. MK wrote the first draft of the manuscript. All authors contributed to the article and approved the submitted version.

## Funding

This research work was supported in part by JST, Moonshot R&D Grant Number JPMJMS2011 (experiment and analysis), JST CREST Grant Number JPMJCR18A1, Japan (system development), JSPS KAKENHI Grant Number JP20K19897, and Tateishi Science and Technology Foundation.

## Conflict of interest

Authors MK and MS were employed by ATR.

The remaining authors declare that the research was conducted in the absence of any commercial or financial relationships that could be construed as a potential conflict of interest.

## Publisher’s note

All claims expressed in this article are solely those of the authors and do not necessarily represent those of their affiliated organizations, or those of the publisher, the editors and the reviewers. Any product that may be evaluated in this article, or claim that may be made by its manufacturer, is not guaranteed or endorsed by the publisher.

## References

[ref1] Althaus P. Ishiguro H. Kanda T. Miyashita T. Christensen H. I. (2004). “Navigation for human-robot interaction tasks” in IEEE International Conference on Robotics and Automation, 2004. Proceedings. ICRA ’04. 2004, 2, 1894–1900. doi: 10.1109/robot.2004.1308100

[ref2] Aslan I. André E. (2017). “Pre-touch proxemics: moving the design space of touch targets from still graphics towards Proxemic behaviors” in Proceedings of the 19th ACM international conference on multimodal interaction ICMI. 17, 101–109. doi: 10.1145/3136755.3136808

[ref3] Bailenson J. N. Blascovich J. Beall A. C. Loomis J. M. (2001). Equilibrium theory revisited: mutual gaze and personal space in virtual environments. Presence Teleoperators Vir. Environ. 10, 583–598. doi: 10.1162/105474601753272844

[ref4] Bailenson J. N. Blascovich J. Beall A. C. Loomis J. M. (2003). Interpersonal distance in immersive virtual environments. Pers. Soc. Psychol. B. 29, 819–833. doi: 10.1177/0146167203029007002, PMID: 15018671

[ref5] Bailenson J. N. Yee N. (2008). Virtual interpersonal touch: haptic interaction and copresence in collaborative virtual environments. Multimed. Tools Appl. 37, 5–14. doi: 10.1007/s11042-007-0171-2

[ref6] Bolt E. Ho J. T. Lesur M. R. Soutschek A. Tobler P. N. Lenggenhager B. (2021). Effects of a virtual gender swap on social and temporal decision-making. Sci Rep-uk 11:15376. doi: 10.1038/s41598-021-94869-z, PMID: 34321591 PMC8319130

[ref7] Bönsch A. Radke S. Ehret J. Habel U. Kuhlen T. W. (2020). The impact of a virtual Agent’s non-verbal emotional expression on a User’s personal space preferences. Proc 20th Acm Int Conf Intelligent Virtual Agents, 1–8.

[ref8] Bönsch A. Radke S. Overath H. Asché L. M. Wendt J. Vierjahn T.. (2018). Social VR: how personal space is affected by virtual agents’ emotions. 2018 Ieee Conf Virtual Real 3d User Interfaces Vr 00, 199–206.

[ref9] Buck L. E. Chakraborty S. Bodenheimer B. (2022). The impact of embodiment and avatar sizing on personal space in immersive virtual environments. Ieee T Vis. Comput. Gr 28, 2102–2113. doi: 10.1109/tvcg.2022.3150483, PMID: 35167460

[ref10] Buck L. E. Rieser J. J. Narasimham G. Bodenheimer B. (2019). Interpersonal affordances and social dynamics in collaborative immersive virtual environments: passing together through apertures. Ieee T. Vis. Comput. Gr 25, 2123–2133. doi: 10.1109/tvcg.2019.2899232, PMID: 30794184

[ref11] Dion K. Berscheid E. Walster E. (1972). What is beautiful is good. J. Pers. Soc. Psychol. 24, 285–290. doi: 10.1037/h0033731 4655540

[ref12] Erp J. B. F. Van Toet A. (2015). Social touch in human–computer interaction. Front. Digital Humanit. 2,:2. doi: 10.3389/fdigh.2015.00002

[ref13] Frisanco A. Schepisi M. Tieri G. Aglioti S. M. (2022). Embodying the avatar of an omnipotent agent modulates the perception of one’s own abilities and enhances feelings of invulnerability. Sci. Rep-uk 12:21585. doi: 10.1038/s41598-022-26016-1, PMID: 36517558 PMC9751071

[ref14] Fusaro M. Lisi M. P. Tieri G. Aglioti S. M. (2021). Heterosexual, gay, and lesbian people’s reactivity to virtual caresses on their embodied avatars’ taboo zones. Sci. Rep-uk 11:2221. doi: 10.1038/s41598-021-81168-w, PMID: 33500486 PMC7838160

[ref15] Gallace A. Girondini M. (2022). Social touch in virtual reality. Curr. Opin. Behav. Sci. 43, 249–254. doi: 10.1016/j.cobeha.2021.11.006

[ref16] Gross A. E. Crofton C. (1977). What is good is beautiful. Sociometry 40:85. doi: 10.2307/3033549

[ref17] Hall E. T. (1966). The hidden dimension. New York: Anchor Books.

[ref18] Hayduk L. A. (1985). Personal space: the conceptual and measurement implications of structural equation models. Canadian J. Behav. Sci. Revue canadienne des sciences du comportement 17, 140–149. doi: 10.1037/h0080132

[ref19] Hinckley K. Heo S. Pahud M. Holz C. Benko H. Sellen A.. (2016). Pre-touch sensing for Mobile interaction. in Proceedings of the 2016 CHI Conference on Human Factors in Computing Systems CHI ‘16. (New York, NY, USA: Association for Computing Machinery), 2869–2881.

[ref20] Hoppe M. Rossmy B. Neumann D. P. Streuber S. Schmidt A. Machulla T.-K. (2020). A human touch: social touch increases the perceived human-likeness of agents in virtual reality. in Proceedings of the 2020 CHI Conference on Human Factors in Computing Systems CHI ‘20. (New York, NY, USA: Association for Computing Machinery), 1–11.

[ref21] Huang A. Knierim P. Chiossi F. Chuang L. L. Welsch R. (2022). Proxemics for human-agent interaction in augmented reality. in Proceedings of the 2022 CHI Conference on Human Factors in Computing Systems CHI, 22, 1–13. doi: 10.1145/3491102.3517593

[ref22] Huisman G. (2016). Social touch technology: a survey of haptic Technology for Social Touch. Ieee T. Haptics 10, 391–408. doi: 10.1109/toh.2017.2650221, PMID: 28092577

[ref23] Huisman G. Bruijnes M. Kolkmeier J. Jung M. Frederiks A. D. Rybarczyk Y. (2014). Touching virtual agents: embodiment and mind. in 9th International Summer Workshop on Multimodal Interfaces (eNTERFACE) Innovative and Creative Developments in Multimodal Interaction Systems. 114–138. doi: 10.1007/978-3-642-55143-7_5

[ref24] Iachini T. Coello Y. Frassinetti F. Ruggiero G. (2014). Body space in social interactions: a comparison of reaching and comfort distance in immersive virtual reality. PLoS One 9:e111511. doi: 10.1371/journal.pone.0111511, PMID: 25405344 PMC4236010

[ref25] Iachini T. Coello Y. Frassinetti F. Senese V. P. Galante F. Ruggiero G. (2016). Peripersonal and interpersonal space in virtual and real environments: effects of gender and age. J. Environ. Psychol. 45, 154–164. doi: 10.1016/j.jenvp.2016.01.004

[ref26] Jo D. Kim K. Welch G. F. Jeon W. Kim Y. Kim K.-H.. (2017). The impact of avatar-owner visual similarity on body ownership in immersive virtual reality. In proceedings of the 23rd ACM symposium on virtual reality software and technology VRST ‘17. (New York, NY, USA: Association for Computing Machinery).

[ref27] Kirby R. Simmons R. Forlizzi J. (2009). COMPANION: a constraint-optimizing method for person-acceptable navigation. Ro-man 2009 -18th Ieee Int Symposium Robot Hum Interact Commun, 607–612.

[ref28] Krekhov A. Cmentowski S. Krüger J. (2019). The illusion of animal body ownership and its potential for virtual reality games. 2019 Ieee Conf Games Cog 00, 1–8.

[ref29] Kruijff E. Riecke B. E. Trepkowski C. Lindeman R. W. (2022). First insights in perception of feet and lower-body stimuli for proximity and collision feedback in 3D user interfaces. Front. Vir. Real 3:954587. doi: 10.3389/frvir.2022.954587

[ref30] Kudry P. Cohen M. (2022). Development of a wearable force-feedback mechanism for free-range haptic immersive experience. Front. Vir. Real 3:824886. doi: 10.3389/frvir.2022.824886

[ref31] Lancaster P. Yang B. Smith J. R. (2017). Improved object pose estimation via deep pre-touch sensing. 2017 Ieee Rsj Int Conf Intelligent Robots Syst Iros, 2448–2455.

[ref32] Lee J.-E. R. Nass C. I. Bailenson J. N. (2014). Does the mask govern the mind?: effects of arbitrary gender representation on quantitative task performance in avatar-represented virtual groups. Cyberpsychol. Behav. Soc. Netw. 17, 248–254. doi: 10.1089/cyber.2013.0358, PMID: 24479529

[ref33] Li R. Almkerk M. van Waveren S. van Carter E. Leite I. (2019). Comparing human-robot proxemics between virtual reality and the real world. In 2019 14th ACM/IEEE international conference on human-robot interaction (HRI)., 431–439.

[ref34] Lisi M. P. Fusaro M. Tieri G. Aglioti S. M. (2021). Humans adjust virtual comfort-distance towards an artificial agent depending on their sexual orientation and implicit prejudice against gay men. Comput. Hum. Behav. 125:106948. doi: 10.1016/j.chb.2021.106948

[ref35] Llobera J. Spanlang B. Ruffini G. Slater M. (2010). Proxemics with multiple dynamic characters in an immersive virtual environment. Acm. Trans. Appl. Percept. Tap. 8:1. doi: 10.1145/1857893.1857896, 12

[ref36] Whitmire E. Benko H. Holz C. Ofek E. Sinclair M. (2018). Haptic revolver: touch, shear, texture, and shape rendering on a reconfigurable virtual reality controller. in Proceedings of the 2018 CHI Conference on Human Factors in Computing Systems CHI, 18, 1–12. doi: 10.1145/3173574.3173660

[ref37] Mejía D. A. C. Saito A. Kimoto M. Iio T. Shimohara K. Sumioka H.. (2021a). Modeling of pre-touch reaction distance for faces in a virtual environment. J. Inf. Proc. 29, 657–666. doi: 10.2197/ipsjjip.29.657

[ref38] Mejía D. A. C. Sumioka H. Ishiguro H. Shiomi M. (2021b). Modeling a pre-touch reaction distance around socially touchable upper body parts of a robot. Appl. Sci. 11:7307. doi: 10.3390/app11167307

[ref39] Mejía D. A. C. Sumioka H. Ishiguro H. Shiomi M. (2023). Evaluating gaze behaviors as pre-touch reactions for virtual agents. Front. Psychol. 14:1129677. doi: 10.3389/fpsyg.2023.1129677, PMID: 36949918 PMC10026528

[ref40] Mello M. Fusaro M. Tieri G. Aglioti S. M. (2021). Wearing same-and opposite-sex virtual bodies and seeing them caressed in intimate areas. Q. J. Exp. Psychol. 75, 461–474. doi: 10.1177/17470218211031557 34169751

[ref41] Mori M. MacDorman K. F. Kageki N. (2012). The Uncanny Valley from the field. IEEE Robot. Autom. Mag. 19, 98–100. doi: 10.1109/mra.2012.2192811

[ref42] Mousas C. Koilias A. Rekabdar B. Kao D. Anastaslou D. (2021). Toward understanding the effects of virtual character appearance on avoidance movement behavior. 2021 Ieee Virtual Real 3d User Interfaces Vr 00, 40–49.

[ref43] Müller J. Rieger L. Aslan I. Anneser C. Sandstede M. Schwarzmeier F.. (2019). Mouse, touch, or Fich: comparing traditional input modalities to a novel pre-touch technique. in Proceedings of the 18th International Conference on Mobile and Ubiquitous Multimedia MUM ‘19. (New York, NY, USA: Association for Computing Machinery).

[ref44] Neyret S. Navarro X. Beacco A. Oliva R. Bourdin P. Valenzuela J.. (2020). An embodied perspective as a victim of sexual harassment in virtual reality reduces action conformity in a later Milgram obedience scenario. Sci Rep-uk 10:6207. doi: 10.1038/s41598-020-62932-w, PMID: 32277079 PMC7148366

[ref45] Novick D. Hinojos L. J. Rodriguez A. E. Camacho A. Afravi M. (2018). Conversational interaction with multiple agents initiated via proxemics and gaze. in Proceedings of the 6th International Conference on Human-Agent Interaction HAI ‘18. (New York, NY, USA: Association for Computing Machinery), 356–358.

[ref46] Nowak K. L. Rauh C. (2005). The influence of the avatar on online perceptions of anthropomorphism, androgyny, credibility, Homophily, and attraction. J. Comput. Mediat. Commun. 11, 153–178. doi: 10.1111/j.1083-6101.2006.tb00308.x

[ref47] Nunez O. J. A. Zenner A. Steinicke F. Daiber F. Krüger A. (2022). Holitouch: conveying holistic touch illusions by combining Pseudo-haptics with tactile and proprioceptive feedback during virtual interaction with 3DUIs. Front. Vir. Real 3:879845. doi: 10.3389/frvir.2022.879845

[ref48] Oh S. Y. Bailenson J. Weisz E. Zaki J. (2016). Virtually old: embodied perspective taking and the reduction of ageism under threat. Comput. Hum. Behav. 60, 398–410. doi: 10.1016/j.chb.2016.02.007

[ref49] Preechayasomboon P. Rombokas E. (2021). Haplets: finger-worn wireless and low-encumbrance Vibrotactile haptic feedback for virtual and augmented reality. Front. Vir. Real 2:738613. doi: 10.3389/frvir.2021.738613

[ref50] Principe C. P. Langlois J. H. (2011). Faces differing in attractiveness elicit corresponding affective responses. Cog. Emot. 25, 140–148. doi: 10.1080/02699931003612098, PMID: 21432661 PMC3269167

[ref51] Rapuano M. Sbordone F. L. Borrelli L. O. Ruggiero G. Iachini T. (2021). The effect of facial expressions on interpersonal space: a gender study in immersive virtual reality. Progresses in Artificial Intelligence and Neural Systems, 477–486. doi: 10.1007/978-981-15-5093-5_40

[ref52] Ratan R. Beyea D. Li B. J. Graciano L. (2020). Avatar characteristics induce users’ behavioral conformity with small-to-medium effect sizes: a meta-analysis of the proteus effect. Media Psychol. 23, 651–675. doi: 10.1080/15213269.2019.1623698

[ref53] Rivu R. Zhou Y. Welsch R. Mäkelä V. Alt F. (2021). When friends become strangers: understanding the influence of avatar gender on interpersonal distance in virtual reality. in Human-Computer Interaction--INTERACT 2021: 18th IFIP TC 13 International Conference (Springer International Publishing), 234–250

[ref54] Ruggiero G. Frassinetti F. Coello Y. Rapuano M. Cola A. S. Di Iachini T. (2017). The effect of facial expressions on peripersonal and interpersonal spaces. Psychol. Res. 81, 1232–1240. doi: 10.1007/s00426-016-0806-x, PMID: 27785567

[ref55] Schulze S. Pence T. Irvine N. Guinn C. (2019). “The Effects of Embodiment in Virtual Reality on Implicit Gender Bias.” in Virtual, Augmented and Mixed Reality. Multimodal Interaction. HCII 2019 Lecture Notes in Computer Science, 361–374. doi: 10.1007/978-3-030-21607-8_28

[ref56] Shell A. K. Pena A. E. Abbas J. J. Jung R. (2022). Novel Neurostimulation-based haptic feedback platform for grasp interactions with virtual objects. Front. Vir. Real 3:910379. doi: 10.3389/frvir.2022.910379

[ref57] Shiomi M. Kubota A. Kimoto M. Iio T. Shimohara K. (2022). Stay away from me: coughing increases social distance even in a virtual environment. PLoS One 17:e0279717. doi: 10.1371/journal.pone.0279717, PMID: 36576927 PMC9797075

[ref58] Shiomi M. Shatani K. Minato T. Ishiguro H. (2018). How should a robot react before People’s touch: modeling a pre-touch reaction distance for a Robot’s face. Ieee Robot. Autom. Lett 3, 3773–3780. doi: 10.1109/lra.2018.2856303

[ref59] Suvilehto J. T. Glerean E. Dunbar R. I. M. Hari R. Nummenmaa L. (2015). Topography of social touching depends on emotional bonds between humans. Proc Nat. Acad Sci. 112, 13811–13816. doi: 10.1073/pnas.1519231112, PMID: 26504228 PMC4653180

[ref60] Suvilehto J. T. Nummenmaa L. Harada T. Dunbar R. I. M. Hari R. Turner R.. (2019). Cross-cultural similarity in relationship-specific social touching. Proc. R. Soc B Biol. Sci. 286:20190467. doi: 10.1098/rspb.2019.0467, PMID: 31014213 PMC6501924

[ref61] Svenstrup M. Bak T. Andersen H. J. (2010). Trajectory planning for robots in dynamic human environments. 2010 Ieee Rsj Int Conf Intelligent Robots Syst, 4293–4298.

[ref62] Sykownik P. Masuch M. (2020). “The experience of social touch in multi-user virtual reality” in Proceedings of the 26th ACM Symposium on Virtual Reality Software and Technology VRST, 20, 1–11. doi: 10.1145/3385956.3418944

[ref63] Szolin K. Kuss D. J. Nuyens F. M. Griffiths M. D. (2022). Exploring the user-avatar relationship in videogames: a systematic review of the Proteus effect. Hum. Comput. Interact., 1–26. doi: 10.1080/07370024.2022.2103419

[ref64] Tajfel H. Billig M. G. Bundy R. P. Flament C. (1971). Social categorization and intergroup behaviour. Eur. J. Soc. Psychol. 1, 149–178. doi: 10.1002/ejsp.2420010202

[ref65] Takayama L. Pantofaru C. (2009). Influences on proxemic behaviors in human-robot interaction. 2009 Ieee Rsj Int Conf Intelligent Robots Syst, 5495–5502. doi: 10.1109/iros.2009.5354145

[ref66] Villa S. Mayer S. Hartcher-O’Brien J. Schmidt A. Machulla T.-K. (2022). Extended mid-air ultrasound haptics for virtual reality. Proc. ACM Hum.-Comput. Interact 6, 500–524. doi: 10.1145/3567731

[ref67] Wackernagel H. (2003). “Ordinary Kriging” in . ed. Wackernagel H. (Berlin, Heidelberg: Springer Berlin Heidelberg), 79–88. doi: 10.1007/978-3-662-05294-5_11

[ref68] Waddell T. F. Ivory J. D. (2015). It’s not easy trying to be one of the guys: the effect of avatar attractiveness, avatar sex, and user sex on the success of help-seeking requests in an online game. J. Broadcast Electron. 59, 112–129. doi: 10.1080/08838151.2014.998221

[ref69] Wieser M. J. Pauli P. Grosseibl M. Molzow I. Mühlberger A. (2010). Virtual social interactions in social anxiety—the impact of sex, gaze, and interpersonal distance. Cyberpsychol. Behav. Soc. Netw. 13, 547–554. doi: 10.1089/cyber.2009.0432, PMID: 20950179

[ref70] Wilcox L. M. Allison R. S. Elfassy S. Grelik C. (2006). Personal space in virtual reality. Acm. Trans. Appl. Percept. Tap. 3, 412–428. doi: 10.1145/1190036.1190041

[ref71] Wu L. Chen K. B. (2022). Examining the effects of gender transfer in virtual reality on implicit gender bias. Hum. Factors. doi: 10.1177/00187208221145264, PMID: 36574504

[ref72] Yee N. Bailenson J. (2007). The Proteus effect: the effect of transformed self-representation on behavior. Hum. Commun. Res. 33, 271–290. doi: 10.1111/j.1468-2958.2007.00299.x

[ref73] Zenner A. Kruger A. (2017). Shifty: a weight-shifting dynamic passive haptic proxy to enhance object perception in virtual reality. Ieee T. Vis. Comput. Gr 23, 1285–1294. doi: 10.1109/tvcg.2017.2656978, PMID: 28129164

[ref74] Zibrek K. Kokkinara E. McDonnell R. (2017). “Don’t stand so close to me: investigating the effect of control on the appeal of virtual humans using immersion and a proximity-based behavioral task” in Proceedings of the ACM symposium on applied perception, 1–11.

[ref75] Zibrek K. Kokkinara E. Mcdonnell R. (2018). The effect of realistic appearance of virtual characters in immersive environments -does the Character’s personality play a role? Ieee T. Vis. Comput. Gr 24, 1681–1690. doi: 10.1109/tvcg.2018.2794638, PMID: 29543183

[ref76] Zibrek K. Niay B. Olivier A.-H. Hoyet L. Pettre J. McDonnell R. (2020). The effect of gender and attractiveness of motion on proximity in virtual reality. ACM Trans. Appl. Percept. 17, 1–15. doi: 10.1145/341998534113222

[ref77] Zibrek K. Niay B. Olivier A.-H. Pettré J. Hoyet L. McDonnell R. (2022). Proximity in VR: the importance of character attractiveness and participant gender. 2022 Ieee Conf Virtual Real 3d User Interfaces Abstr Work Vrw 00, 672–673. doi: 10.1109/vrw55335.2022.00187

